# Worsening suicidal ideation and prolonged adverse event following psilocybin administration in a clinical setting: case report and thematic analysis of one participant's experience

**DOI:** 10.1192/bjo.2024.768

**Published:** 2024-12-10

**Authors:** Mourad Wahba, Caroline Hayes, Maartje Kletter, R. Hamish McAllister-Williams

**Affiliations:** Wolfson Research Centre, Cumbria, Northumberland, Tyne and Wear NHS Trust, Newcastle upon Tyne, UK; and Campus for Ageing and Vitality, Newcastle University, Newcastle upon Tyne, UK; Cumbria, Northumberland, Tyne and Wear NHS Trust, Newcastle upon Tyne, UK; School of Health Sciences, Division of Nursing, Midwifery & Social Work, University of Manchester, Manchester, UK; Translational and Clinical Research Institute, Faculty of Medical Sciences, Newcastle University, Newcastle upon Tyne, UK; and Cumbria, Northumberland, Tyne and Wear NHS Foundation Trust, Newcastle upon Tyne, UK

**Keywords:** Psilocybin, suicidality, adverse event, psychedelics, case report

## Abstract

**Background:**

Psilocybin is being investigated as a treatment for a myriad of disorders, including treatment-resistant depression. The main focus has been on positive effects, with little attention paid to negative outcomes, especially in clinical settings. Quantitative methodology limits further exploration of such events and can also miss improvements not captured on rating scales.

**Aims:**

To highlight potential adverse events of psilocybin and underline limits of quantitative methodology, calling for process evaluations alongside clinical trials.

**Case presentation:**

This is a case of a participant in a phase 2b clinical trial of psilocybin for treatment-resistant depression who presented with increased suicidal ideation and a prolonged period of severely restricted eating following administration, leading to a period of destabilisation and a need for support. Despite the difficulties encountered and the participant's limited improvement on rating scales, she found the experience to have been helpful and led her to make changes to her life which she found beneficial. She described her experience in a written account to the authors.

**Method:**

The case was summarised and the written account was thematically analysed and synthesised into a logic model.

**Conclusions:**

Psilocybin could lead to temporary worsening of suicidal ideation and instigate prolonged adverse events that outlast its acute effects. Paradoxically, it could simultaneously lead to an improvement in functional outcomes which is not clear on depression rating scales. This calls for a qualitative exploration of serious adverse events and participant accounts to deepen our understanding of the psilocybin experience and its different outcomes.

Psychedelics are gathering momentum as compounds of potential therapeutic value within the scientific community,^1–3^ in the mainstream media^4,5^ and among patients searching for new and promising treatments, as more accounts of benefits are published.^6^ This is a welcome change from the predominantly negative narrative that was associated with psychedelics prior to the recent resurgence of interest in their therapeutic properties, and it is supported by promising results from early clinical trials.^7–11^ Larger studies needed to validate these results are currently in development, and a phase 3 programme for treatment-resistant depression is currently underway. However, there is a risk of replacing the stigma and historically predominantly negative narrative with an equally misleading and overwhelmingly positive narrative. This has already been explored within the scientific community^12–14^ and is a factor that needs to be considered when working with these substances, to provide a balanced view of their benefits and harms, with a focus on science rather than media hype.

This case report was written on the basis of the authors’ involvement with COMP001, COMPASS Pathways’ phase 2b clinical trial of the investigational drug COMP360 (COMPASS Pathways’ proprietary synthetic psilocybin formulation) for treatment-resistant depression,^11^ and COMP004, a long-term follow-up study (Fig. 1). In COMP001, psilocybin (1, 10 or 25 mg) was administered in a double-blind fashion under therapist supervision in a comfortable, non-clinical environment. This was preceded by two sessions of preparation, which included psychoeducation and building rapport, and followed by two sessions of integration, during which participants shared their experience with the therapist.

**Fig. 1:**
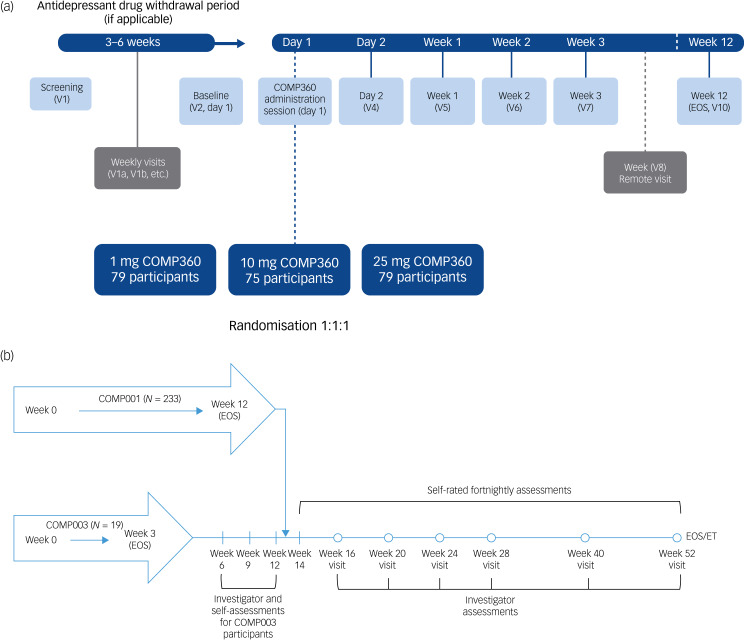
(a) Overview of COMP001. (b) Overview of COMP004, which included participants from two trials (COMP001, and COMP003). Participants were followed up for a year in total. V, visit, where V1a, V1b, etc. refer to visits between V1 and V2 which supported antidepressant withdrawal and preparation; EOS, end of study; ET, early termination.

This participant was one of 12 who experienced serious adverse events during COMP001, having experienced two herself. It was felt that her case needed highlighting for several reasons:

- it demonstrates that suicidal ideation can worsen following psilocybin administration, even within a supportive clinical setting;
- it illustrates the complexity of the experience of patients with difficult-to-treat depression and demonstrates that participants can have prolonged adverse reactions following psilocybin experiences;
- it suggests that some patients classed as ‘non-responders’ can in fact have positive outcomes not expressed in the data, highlighting the limitations of quantitative studies in capturing paradoxical positive changes in affect that are not necessarily reflected in changes on depression rating scales.

## Method

The participant was approached by the authors (M.W. and C.H.) to suggest writing the report. She gave her consent and offered to write a comprehensive account of her experience. This was analysed by M.K. using a thematic analysis,^15^ themes were developed based on similarities, and identified themes were synthesised into a logic model.^16^ The participant was involved throughout the process to ensure accuracy, and the manuscript was shared with COMPASS Pathways, the trial sponsor, to enable them to provide relevant trial data and to comment on the manuscript before submission. Ethical approval was not required for this report.

## Case presentation

Throughout this report, participant details have been changed to de-identify the individual. She will be referred to as ‘Zakara’.

Zakara^17^ had a history of recurrent depression which started in 2012 and continued throughout her life, with the current episode being of 2.5 years duration with no response to citalopram or sertraline at adequate dose and duration. She had a history of chronic fatigue syndrome in 2017 and of meningitis as a baby, which had resolved without sequalae. Otherwise, she had no significant physical health history.

The Mini International Neuropsychiatric Interview confirmed a primary diagnosis of moderate depressive disorder (recurrent) and social anxiety disorder. She reported suicidal thoughts at the time of screening, both in the past year and throughout her life, albeit without intent, and cited her family as a protective factor. She reported thinking about suicide as a fantasy of escape from her emotional pain and, despite having no intent to hurt herself, she had visited the train tracks to ‘see what they were like’ when she was particularly low in mood, in the year before dosing.

This did not meet the criteria for significant suicide risk as defined by (a) suicidal ideation as endorsed on items 4 or 5 on the Columbia Suicidality Severity Rating Scale within the past year, screening, or at baseline; or (b) suicidal behaviours within the past year; or (c) clinical assessment of significant suicidal risk during subject interview.

Her vital signs and electrocardiogram were normal, and there were no significant clinical abnormalities in her work-up. Other rating scales included the Mclean Screening Instrument for Borderline Personality Disorder, on which she scored 2, well below the cut-off to suggest a diagnosis of emotionally unstable personality disorder; and the Adult ADHD Self-Report Scale, where she scored 17, indicating a possibility of comorbid ADHD. At baseline, her Hamilton Rating Scale for Depression score was 25, indicating severe depression, and her Montgomery–Åsberg Depression Rating Scale (MADRS) score was 33, indicating moderate depression.

On dosing day, she was randomised to 25 mg COMP360 (administered double-blind with respect to dose). After a good start, Zakara started to struggle and found the latter half of the day very distressing. Following the experience, she reported feeling that her existence was ‘pointless’ and her death by suicide was ‘inevitable’. She had no intent to act on these thoughts and had support from her mother for the night, with 2 × 1 mg of lorazepam provided to be used as required.

**Fig. 2:**
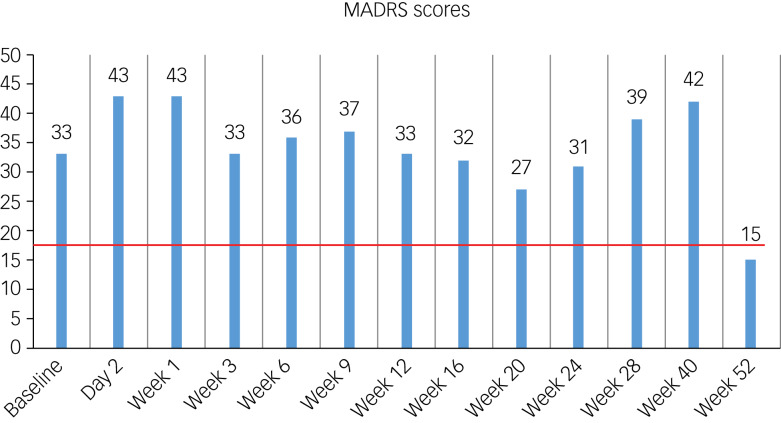
Montgomery–Åsberg Depression Rating Scale (MADRS) scores throughout COMP001 and COMP004. These are independently rated MADRS scores throughout involvement in the study. The line in red represents the line of response, defined as a 50% reduction from baseline. This was reached shortly after starting duloxetine.

The following morning (1 July 2021), Zakara reported feeling ‘hung over’, described feeling ‘rocky’ and was unable to eat breakfast owing to persistent nausea. She also reported having thoughts of jumping out of the window the night before. She explained that her wish to be dead was now more intense and her suicidal thoughts now constant and more difficult to control, but she had no intent to follow through, with her protective factors still in place. She was offered the first of three additional integration sessions which were spread across the rest of the study and a handover was given to the local crisis team for support if required. At this point her MADRS was 43 (Fig. 2), indicating severe depression. Four days post-dosing, Zakara contacted the team reporting a significant deterioration in her mood, reporting it was ‘the worst she'd ever felt’. She was experiencing panic attacks, disrupted sleep and an inability to eat due to persistent nausea. She was also struggling with an increase in her suicidal thoughts, with some intent to act on them, although this fluctuated. She had impulses to go to the train tracks near her home, which she did, but only so ‘her brain would shut up’, as she was having thoughts of wanting to go. Zakara made it clear at the time that she had no intent of taking her life when she visited the tracks. She did, however, consider taking an overdose of her prescribed propranolol (see Table 1 for all medications prescribed). She reached out to ‘Breathing Space’ (a free, confidential, phone and webchat service for anyone in Scotland over the age of 16 experiencing low mood, depression or anxiety), and the study team referred her to the crisis team, who supported her until her next appointment (week 1). This was the first serious adverse event, consisting of suicidal ideation with some intent.

**Table 1:** Medications used throughout COMP001 and COMP004

Drug	Indication	Dose	Time point	Duration
Propranolol	Anxiety	10–20 mg as required (max. 3 times daily).	From screening	Until increase
		40 mg 3 times daily	~5 weeks post-dosing	Ongoing at end of long-term follow-up
Lorazepam	Anxiety	1 mg as required	1 day post-dosing	1 day
Diazepam	Anxiety and insomnia	5 mg as required	4 days post-dosing	9 days
Promethazine	Insomnia	25–50 mg	10 days post-dosing	10 days
Trazodone	Anxiety	50–100 mg	7 weeks post-dosing	7 days
Quetiapine	Anxiety	25–50 mg	~12 weeks post-dosing	Ongoing at end of long-term follow-up
Duloxetine	Depressive symptoms	30 mg	~50 weeks post-dosing	Ongoing at end of long-term follow-up

max., maximum.

Despite her struggles, Zakara was able to go to work 6 days post-dosing. She was supported with diazepam, which she found very helpful. She continued to struggle with low mood, but her suicidal thoughts were improving. One week post-dosing, her presentation was less acute and she reported feeling better although still worse than her baseline. Her panic attacks had stopped.

Two weeks post-dosing, Zakara continued to struggle, although she started making changes to her daily life. She joined a fitness club and reported that her suicidal thoughts were less constant. By 6 weeks post-dosing, Zakara was able to do all her usual work and activities (such as seeing friends and exercising) despite her low mood. She was looking to change to a more fulfilling job and felt that her concentration on things that she enjoyed was better than before dosing, suggesting some functional improvement despite MADRS scores remaining high.

Nine weeks post-dosing, Zakara had her second serious adverse event. Distressed by personal circumstances, which included friends leaving the city and family being away, she contemplated taking an overdose, counted the medication she had at home, and started writing a suicide note but stopped of her own accord. Twelve weeks post-dosing (the end of the COMP001 study), she felt she was managing better overall and was able to recognise a distance between her and her negative thoughts. She had no suicidal ideation and had significantly reduced death wishes, but she still struggled with her sleep and appetite. Zakara was then enrolled in COMP004.

Twenty-four weeks post-dosing, improvement continued as she reported being ‘generally well’. She had started a new job and was happy with the changes she made in her life. Her nausea and consequent inability to eat persevered for around 9 months before starting to improve and only ceased being of concern around 1 year after dosing (weight measurements were not included as per Zakara's preference). This was severe and significant in its impact, with her being unable to eat more than 300 calories per day, leading to hair thinning, rapid weight loss and fainting at one point. She did have a history of diet restriction a few years prior to enrolment, which she felt was ‘activated’ after the experience. She described not restricting intentionally but rather being physically unable to eat owing to severe nausea and her anxiety. There was a concern over anorexic cognitions returning a few months after dosing, given that she reported ‘having an internal voice that told her what she could and couldn't eat’, which was easier to ignore with the higher dose of quetiapine (these were not hallucinations).

One year after dosing, she felt she was in a better place overall, with her mood being more stable and appetite back to normal. She felt that despite the difficulties she encountered, the trial had been a positive experience for her. She summarised her improvement in a small, but meaningful change she had noticed, which was that she had brushed her teeth every day since dosing.

## Thematic analysis

‘I don't want my experience to be used as a warning against the use of psilocybin therapeutically. But I also don't want it to be brushed aside as a fluke, or as a non-responder (Oh boy, did I respond)’

Twenty-nine themes were identified from the written account provided by Zakara, the most relevant of which are presented in Table 2 below (see Supplementary material available at https://doi.org/10.1192/bjo.2024.768 for all themes). Themes were categorised as per logic model components: inputs, processes, outputs, outcomes and impact, to provide a context for the themes as portrayed in the original text.

**Table 2:** Overview of relevant themes

Theme	Summary and quotes
**Inputs** Themes here relate to available resources pre-dosing that potentially influenced outcomes	
Expectations prior to participation	Expectations of what the outcome of dosing would be like (a new start). *‘I understood the effects of psilocybin to be like fresh-fallen snow over old footprints that one could tread new paths on. I concluded that for me it was more revealing than resetting. It wasn't a snowfall that filled in some bad paths, it was a hurricane that blew away the topsoil to reveal the rotting detritus underneath.’*
Perceived positivity of information provided	The information provided before the dosing was perceived to be overly positive. Difficulty imagining what a psychedelic experience would entail led to difficulties interpreting potential negative side-effects. *‘I do remember a list of side-effects listing something like “worsening mental health” under rare, with a note in brackets saying, “this wasn't seen in similar studies”[…]. going into the dosing I did have a realistic expectation that things might not get better, and I could come out of this having received the 1 mg dose and be more or less in the same position. I had no notion that it could actively make things worse.’*
**Process** Themes here relate to processes during the dosing session.	
Symbolic experiences during dosing	Various symbolic experiences during dosing were described, including a pattern that was deemed to be inescapable. *‘With the same dizzying feeling the moment one perceives the secret of an optical illusion or a trick of perspective, the universe was merely the smallest iteration of an even larger pattern. There was nothing but the pattern. No escape. Even the most vast and momentous of things was just an insignificance to the pattern.’*
Emotions experienced during dosing	Various emotions were experienced during dosing. *‘A great, inky smoke began to billow out of my mouth. It felt viscous, like soot, and was toxic and painful to emotionally vomit up, but what a relief I felt! […] it was everything I had ever bottled up or locked away; every bit I had cut off when I was just a bit too big or loud for someone's liking; every emotion I had ever been told was bad or shameful;[…] All of it was finally expressing itself […]. They asked me to do some breathing exercises to calm down and regain control. And I did […]. The black smoke, which had been slowly convening into a slightly ungainly cloud on the ceiling was sucked back into my mouth and shoved haphazardly back inside.’*
Difficulties expressing oneself	Zakara experienced difficulties expressing herself during dosing and felt she couldn't communicate difficulties sufficiently with those in the room. *‘I remember being afraid to reach out for help from the therapist because I had no idea how to explain what was going on for one, but also I was ashamed I had managed to mess up so badly.’*
The role of music	The music played a big part in Zakara's description of the experience, with music affecting her emotions. *‘I can't remember which came first, but I began to have an aversion to the music itself and the critical voice had a lot to say about it: “This is all because of the music. It's clearly designed to instigate manufactured emotional responses from participants.”’*
Physical effects	Various physical effects, including nausea were described. *‘[…] I realised that I couldn't feel my body. I knew on some level that I had pins and needles in my foot, but when I touched my leg it just felt like a foreign object with no sensation’* *‘I remember taking note of the intricacies of the pattern, how I felt sick whenever we passed through a veil.’*
**Output** Themes here relate to immediate changes that occurred after dosing	
Waking up	After dosing, Zakara felt she had woken up from a daze, which ultimately led to the realisation that things needed to change. *‘[…] things felt real for the first time in so long I can't even remember, not just a haze that often felt like a simulation.’* *‘[…] I no longer think of my mental illness as a parasite or shrapnel in my brain. Now it is a part of my mind that has been repressed and acting to be heard without the privilege of access to the ability to speak. It's no longer the malignant tumour that needs excised; it's the bullied teenager who's locked themselves in their room to self-harm when they're just wanted down for family dinner.’*
Suicidality	Zakara contemplated suicide after dosing. *‘*[relating to the pattern] *I felt as though I had discovered a sick truth of life: That no matter how hard you try to climb to better things, you will never succeed; and if you don't work, thing will spiral into deeper and darker depression. The only way out is death. Now that I knew that was the truth, I was cursed. To try to think differently was to pretend and to live a pitiful lie. But to accept it was a pain that could only be cured by suicide. It was so antithetical to my conscious outlook on life.’*
Disordered eating	Zakara experienced nausea during and after dosing and struggled with disordered eating for around a year after dosing. *‘Unfortunately, the part of me that woke up from the coma was also the part of me that struggled with disordered eating – particularly restricting – and this was triggered by the anxiety-driven anorexia and also by multiple people in my personal life complimenting me on my weight loss.’*
Feeling of failure	Zakara felt like she disappointed everyone and failed during trial participation. *‘Although I did my best it was hard to fully engage with some of the prescribed elements of the post-dose therapy. I had an inescapable “knowledge” of the meaningless of life […] Thinking of what activities and mindfulness practice I could do felt like pretending, like trying to salvage tatters of benefit from my massive failure. Although they tried I never really did manage to fully internalise their assurance I hadn't done anything wrong. It's always just felt like consolation.’*
Anxiety	Zakara experienced anxiety after trial participation and was too afraid to close her eyes following the experience, and she experienced fear of the future. *‘Whereas previously I'd always been a heavily future-focused person, I developed a strong fear of the future after the dose.’*
**Outcome** Themes here relate to medium-term outcomes, which started to appear after the sub-acute, post-dosing effects subsided.	
Realisation of a need for change	Trial participation showed Zakara she couldn't go on as she was, and realised that she needed help to change. *‘This need for comfort motivated me to make lots of changes in the aftermath of the dose. It felt as though a part of me had been woken up from a coma I hadn't even realised they'd fallen into when I left Uni the first time, and they were horrified how different their life was to what they had wanted.’*
Realisation of possibility of happiness	The experience showed Zakara that happiness was possible, and during one part she was filled with a goodness she hadn't experienced since they were a child. *‘[…] before the pattern, there was lots of themes and imagery from my childhood that left me with a feeling of goodness I haven't felt since I was seven. Life is currently about trying to find even just a drop of that feeling again and using it to regrow myself. I'm exploring some old hobbies and interests that inspire it.’*
Improvements in negative outputs	Over time, the immediate negative outputs reduced as Zakara learned to live in the present and was able to develop habits she was unable to develop previously, including going to the gym. *‘I kept up the gym consistently for a record time in my life (nearly a whole year!) and after an unintentional, slightly protracted break, due to many factors, I started back recently and am enjoying it still. In addition, I recently began using a bike to commute to work which has been a fun change for me.’*
**Impact** This theme relates to the ultimate impact of the experience on Zakara's life.	
New strategy going forward	Trial participation has provided Zakara with context about what she was experiencing, and this has helped her to develop a new strategy in how to cope going forward. *‘I quit[…] got a job that was challenging and interesting to me; I joined a gym and was able to attend regularly for the first time in my life[…] I gutted my belongings and decided I would only surround myself with things that brought me joy or were immediately useful to me[…] I was even able to manage small amounts of mindfulness meditation, something I had always found extremely challenging and often even distressing[…] Life was beginning to feel like a cold shower – somewhat unpleasant, but invigorating – enlivening.’* ‘*It's probably a puzzle I will be putting together until I die, but whereas before I was struggling with countless pieces that had no context with no idea what I was even trying to put together, the trial experience gave me a glimpse of the picture on the front of the box. That glimpse has already helped me make sense of huge chunks of the puzzle, both at the time and since, and has enabled me to create a strategy on how to tackle the rest going forwards.’*

## Discussion

This case report illustrates that deterioration in mood and increased suicidal ideation can occur following psilocybin administration in the clinical setting. This is well reported in the non-clinical literature, where some recreational users of psilocybin mushrooms report the experience to have been among the most challenging of their lives,^18^ with a recent survey of psychedelics users highlighting that 8.9% of people reported impairment that lasted longer than a day, 4.6% reported severely impaired ability to function, and 6.7% reported thoughts or attempts of hurting themselves or others.^19^ It is not unusual for depression treatments to be associated with an increase in suicidal ideation early in treatment,^20^ and it is well established that within psychotherapy, clients can experience destabilisation before improvement, especially when confronting difficulties they had been avoiding.^21^ Using the same concept, and given that psychedelics are hypothesised to leave individuals feeling more open and less avoidant of internal processes, experiencing emotions that were previously numbed,^22^ it is not unexpected that periods of mood instability may occur after dosing.

Zakara's experience also calls attention to the possibility of prolonged adverse events following psilocybin therapy, given that she struggled with disordered eating that persisted for months following the experience. This has not been clearly captured in the clinical literature so far.

Another aspect to be highlighted is the limitations of quantitative methodology in capturing longitudinal change. Despite falling into the category of being a non-responder,^11^ and not showing a clinical improvement response on rating scales until 1 year after dosing (shortly after starting duloxetine), Zakara was very clear about the earlier benefit of the experience in helping her reconnect with herself and ‘waking up’ to the reality she was living in but distracting herself from. A change in job, a focus on physical exercise and looking after her health, an increased awareness of her internal state and, above all, small daily changes in self-care all clearly show a significant impact on her day-to-day life, despite this not being reflected in her mood rating scale scores. The same concept applies to adverse events, which are not completely captured using quantitative methods. McNamee and others have highlighted the underrepresentation and inadequate investigation of adverse events and the importance of qualitative exploration.^23^ One aspect underlined through qualitative exploration in this instance is the role of expectation in the treatment process. It has been argued that expectancy based on ‘media hype’ may inflate effects in clinical trials.^12^ This case illustrates the possibility that an anticipation of a ‘reset’ or ‘reboot’^24,25^ may in fact have a detrimental effect if the experience is unexpected and the ‘reset’ is not attained.

Finally, Zakara's description of her changes post-dosing suggest a reduction in avoidance of negative emotions and having a fuller experience of her mental and emotional life, in line with suggestions that psilocybin reduces experiential avoidance.^26^ The lack of immediate response, however, raises the question of whether other elements are necessary, e.g. emotional breakthrough, which was correlated with improvement in this trial.^27^ Zakara's responses on the Emotional Breakthrough Inventory suggest that whereas she did face difficult emotions, she did not experience a sense of emotional release, nor did she feel she had an emotional breakthrough or a sense of closure on emotional problems. This could correlate phenomenologically with the salient moment in Zakara's experience, where she felt there was a ‘black cloud’ coming out, and feeling a sense of catharsis that was interrupted.

## Recommendations

Context plays an important part in psilocybin therapy,^28^ and a significant aspect of this is preparation. It is therefore recommended that managing expectation and providing information on possible outcomes following psilocybin therapy is well covered in the preparation phase. This should include the potential for destabilisation and increased suicidal ideation and the need for further support following the session. This allows participants to be well informed and better prepared for any potential after-effects that may occur, and it will help to reduce the idea of a ‘magic treatment’ with very limited adverse events. It remains unclear what the long-term effects of psilocybin are, and long-term follow-up will be necessary to gain a better understanding of these effects; this would also provide an opportunity for aftercare for those who require it.

In addition, given the richness of the psychedelic experience and the limitations of quantitative methodology in capturing this, it would be of value to include process evaluation and qualitative analysis of participant accounts, e.g. at baseline, the point of primary outcome and the end of the trial, with a special focus on those who suffered serious adverse events. This would help to highlight vulnerabilities and identify circumstances associated with such events and refine the screening and administration process. This is further supported by the Medical Research Council's guidance for complex interventions in care,^17^ which recommends conducting a process evaluation alongside implementation of such interventions. Psilocybin therapy is considered a complex intervention, given the number of components involved and the significance of context and delivery for the outcome.^28^

In conclusion, this case illustrates that psilocybin administration could be followed by a worsening in suicidal ideation and prolonged adverse events that long outlast its acute effects. Paradoxically, it may lead to an improvement in functional outcome that is not clear on depression rating scales. As this is a single case report, no conclusions can be drawn, and further assessment is required to understand this phenomenon. Another limitation to note is the likelihood that Zakara had guessed she had received an active dose given the intensity of the experience and was thus unblinded; this could have affected her long-term outcomes.

## Supporting information

Wahba et al. supplementary material 1 Wahba et al. supplementary material

Wahba et al. supplementary material 2 Wahba et al. supplementary material

## Data Availability

Data availability is not applicable to this article as no new data were created or analysed in this study. Zakara's account is available; however, it will only be shared at her discretion owing to its personal nature.

## References

[1] McClure-Begley TD, Roth BL. The promises and perils of psychedelic pharmacology for psychiatry. Nat Rev Drug Discov 2022; 21(6): 463–73. 35301459 10.1038/s41573-022-00421-7

[2] Vollenweider FX, Preller KH. Psychedelic drugs: neurobiology and potential for treatment of psychiatric disorders. Nat Rev Neurosci 2020; 21(11): 611–24. 32929261 10.1038/s41583-020-0367-2

[3] Ko K, Kopra EI, Cleare AJ, Rucker JJ. Psychedelic therapy for depressive symptoms: a systematic review and meta-analysis. J Affect Disord 2023; 322: 194–204. 36209780 10.1016/j.jad.2022.09.168

[4] Jacobs A. The psychedelic revolution is coming. Psychiatry may never be the same. *New York Times*, 5 Sep 2021.

[5] Prideaux E. The worldview-changing drugs poised to go mainstream. *BBC*, 7 Sep 2021.

[6] Carpenter DE. Saved by psychedelics: after traditional methods fail, ayahuasca heals a deep emotional trauma. *Forbes*, 27 Jan 2020.

[7] Slomski A. Psilocybin for treatment of alcohol use disorder. JAMA 2022; 328(13): 1288. 10.1001/jama.2022.15436 36194230

[8] von Rotz R, Schindowski EM, Jungwirth J, Schuldt A, Rieser NM, Zahoranszky K, et al. Single-dose psilocybin-assisted therapy in major depressive disorder: a placebo-controlled, double-blind, randomised clinical trial. eClinicalMedicine 2023; 56: 101809. 36636296 10.1016/j.eclinm.2022.101809 PMC9830149

[9] Mitchell JM, Bogenschutz M, Lilienstein A, Harrison C, Kleiman S, Parker-Guilbert K, et al. MDMA-assisted therapy for severe PTSD: a randomized, double-blind, placebo-controlled phase 3 study. Nat Med 2021; 27(6): 1025–33. 33972795 10.1038/s41591-021-01336-3 PMC8205851

[10] Carhart-Harris R, Giribaldi B, Watts R, Baker-Jones M, Murphy-Beiner A, Murphy R, et al. Trial of psilocybin versus escitalopram for depression. N Engl J Med 2021; 384(15): 1402–11. 33852780 10.1056/NEJMoa2032994

[11] Goodwin GM, Aaronson ST, Alvarez O, Arden PC, Baker A, Bennett JC, et al. Single-dose psilocybin for a treatment-resistant episode of major depression. N Engl J Med 2022; 387(18): 1637–48. 36322843 10.1056/NEJMoa2206443

[12] Butler M, Jelen L, Rucker J. Expectancy in placebo-controlled trials of psychedelics: if so, so what? Psychopharmacology 2022; 239(10): 3047–55. 36063208 10.1007/s00213-022-06221-6 PMC9481484

[13] Yaden DB, Potash JB, Griffiths RR. Preparing for the bursting of the psychedelic hype bubble. JAMA Psychiatry 2022; 79(10): 943–4. 36044208 10.1001/jamapsychiatry.2022.2546

[14] Noorani T, Martell J. New frontiers or a bursting bubble? Psychedelic therapy beyond the dichotomy. Front Psychiatry 2021; 12: 727050. 34566724 10.3389/fpsyt.2021.727050 PMC8460766

[15] Braun V, Clarke V. Using thematic analysis in psychology. Res Psychol 2008; 3(2): 77–101.

[16] W.K. Kellogg Foundation. Using Logic Models to Bring Together Planning, Evaluation and Action. W.K. Kellogg Foundation, 2004.

[17] Skivington K, Matthews L, Simpson SA, Craig P, Baird J, Blazeby JM, et al. A new framework for developing and evaluating complex interventions: update of Medical Research Council guidance. BMJ 2021; 374: n2061. 34593508 10.1136/bmj.n2061 PMC8482308

[18] Carbonaro TM, Bradstreet MP, Barrett FS, MacLean KA, Jesse R, Johnson MW, et al. Survey study of challenging experiences after ingesting psilocybin mushrooms: acute and enduring positive and negative consequences. J Psychopharmacol 2016; 30(12): 1268–78. 27578767 10.1177/0269881116662634 PMC5551678

[19] Simonsson O, Hendricks PS, Chambers R, Osika W, Goldberg SB. Prevalence and associations of challenging, difficult or distressing experiences using classic psychedelics. J Affect Disord 2023; 326: 105–10. 36720405 10.1016/j.jad.2023.01.073 PMC9974873

[20] Stone M, Laughren T, Jones ML, Levenson M, Holland PC, Hughes A, et al. Risk of suicidality in clinical trials of antidepressants in adults: analysis of proprietary data submitted to US Food and Drug Administration. BMJ 2009; 339: b2880. 19671933 10.1136/bmj.b2880 PMC2725270

[21] American Psychological Association (APA). Understanding Psychotherapy and How it Works. APA, 2023 (https://www.apa.org/topics/psychotherapy/understanding).

[22] Gorman I, Nielson EM, Molinar A, Cassidy K, Sabbagh J. Psychedelic harm reduction and integration: a transtheoretical model for clinical practice. Front Psychol 2021; 12: 645246. 33796055 10.3389/fpsyg.2021.645246 PMC8008322

[23] McNamee S, Devenot N, Buisson M. Studying harms is key to improving psychedelic-assisted therapy—participants call for changes to research landscape. JAMA Psychiatry 2023; 80: 411–2. 10.1001/jamapsychiatry.2023.0099 36988924

[24] Gallagher J. Magic mushrooms can ‘reset’ depressed brain. *BBC News*, 14 Oct 2017.

[25] Siddique H. Magic mushrooms ‘reboot’ brain in depressed people – study. *Guardian*, 13 Oct 2017.

[26] Zeifman RJ, Wagner AC, Watts R, Kettner H, Mertens LJ, Carhart-Harris RL. Post-psychedelic reductions in experiential avoidance Are associated with decreases in depression severity and suicidal ideation. Front Psychiatry 2020; 11: 782. 10.3389/fpsyt.2020.00782 PMC7438781 32903724

[27] Goodwin G. Predicting depression outcomes through the influence of therapeutic alliance and the psychedelic experience using path modelling in a phase IIb randomised controlled trial of COMP360 psilocybin therapy. Neuropsychopharmacology 2022; 47: 200.

[28] Carhart-Harris RL, Roseman L, Haijen E, Erritzoe D, Watts R, Branchi I, et al. Psychedelics and the essential importance of context. J Psychopharmacol 2018; 32(7): 725–31. 29446697 10.1177/0269881118754710

